# Vitamin D and Its Relationship With Breast Cancer: An Evidence Based Practice Paper

**DOI:** 10.5539/gjhs.v7n1p261

**Published:** 2014-09-28

**Authors:** Jawad Obaidi, Eyad Musallam, Hamzah Mohammad Al-ghzawi, Saleh Nasser Azzeghaiby, Ibrahim Nasir Alzoghaibi

**Affiliations:** 1 Al-albait university, Faculty of Nursing, Al mafraq, Jordan; 2 Nursing department, Al-Farabi College, Riyadh 11514, Saudi Arabia; 3 Faculty of Nursing, AL al-Bayt University, Mafraq 25113, Jordan; 4 Al-Farabi College, Riyadh, Saudi Arabia

**Keywords:** breast, cancer, vitamin D, neoplasm

## Abstract

**Background:**

In oncology research fields, vitamin D has emerged as the most fruitful issue. The previous decade witnessed intensive efforts in connecting vitamin D with risk reduction and progression of various epithelial cancers, especially, breast cancer.

**Purpose:**

To evaluate the relationship between vitamin D levels and breast cancer.

**Method:**

A comprehensive search of several electronic databases was conducted in Pub Med, MEDLINE, CINAHL, in addition to, web search engine “Google” for abstracts, in order to determine the relationship between vitamin D and breast cancer.

**Results:**

It was found that an increased serum level of vitamin D is associated with decreased risk of breast cancer.

**Conclusion:**

It was concluded that vitamin D plays a significant role in protection of breast cancer.

## 1. Introduction

Vitamin D is a steroid hormone that affects on every cell in the body. Receptors that respond to vitamin D have been found in almost on all type of the body cell, and this helps to clarify why it is has this strong impact on so many diseases (John et al., 2007).

In the oncology research field, vitamin D has emerged as the most fruitful issue in the previous decade with effort connecting it with risk reduction and progression in various epithelial cancers especially in breast cancer.

The relationship between vitamin D and its role in cell growth and differentiation was first established in 1981 when Abe and his colleagues (Abe et al., 1981) studied the role of the vitamin D metabolite, 1α, 25-dihydroxyvitamin D_3_, on mouse myeloid cells. Their research determined that vitamin D is involved in the differentiation of bone marrow cells. These new and exciting findings led to researches that investigated the relationship between vitamin D levels and the potenti al impact on cancer.

In this paper, pertinent studies will be reviewed in a way that leads to an exploration of the relationship between vitamin D levels and breast cancer. The relationship between vitamin D levels and breast cancer will be evaluated, posing the question, “Are women with breast cancer who are vitamin D deficient more likely to have disease progression than women with breast cancer who are not vitamin D deficient?” then the review will classify the available evidence using Johns Hopkins Nursing Evidence-Based Practice Model and Guidelines (Newhouse et al., 2007) as in Table 1, and also the review will identify areas where further research is needed.

**Table 1:** Johns Hopkins Nursing Evidence-Based Practice Model and Guidelines

Level of evidence	Criteria	Quality Rating	Criteria
Level I	Systematic review of relevant randomized controlled trials (with meta-analysis where possible).	A	Consistent results, sufficient sample size, adequate control, and definitive conclusions; consistent recommendations based on extensive literature review that includes thoughtful reference to scientific evidence.
Level II	One or more well designed randomized controlled trials.	B	Reasonably consistent results, sufficient sample size, some control, and fairly definitive conclusions; reasonably consistent recommendations based on fairly comprehensive literature review that includes some reference to scientific evidence
Level III	Well designed nonrandomized controlled trials OR from well designed cohort or case-control analytical studies, preferably multicenter or conducted at different times.	C	Little evidence with inconsistent results, insufficient Sample size, conclusions cannot be drawn.

## 2. The Relationship Between Vitamin D and Breast Cancer

There are many studies that investigated the relationship between vitamin D levels and breast cancer, with slight variations from study to study. In a study by Yao et al. (2011), it was found that increased serum levels were associated with decreased risk of breast cancer. Specifically, they found that those with sufficient levels had a 63% reduction in odds of breast cancer.

Goodwin et al. (2009) conducted a study on 512 women with early breast cancer diagnosed 1989 to 1996. Vitamin D levels were measured in stored blood. Clinical, pathologic, and dietary data were accessed to examine prognostic effects of vitamin D. It was concluded that those with decreased levels of vitamin D had an increased risk of distant recurrence and death compared to those with sufficient levels.

Palmieri et al. (2006) conducted a study to clarify the role of vitamin D in breast cancer progression by comparing the levels of serum vitamin D in patients with early diagnosis and in those with advanced breast cancer. It was found that a relationship between vitamin D levels and breast cancer, though it was not causal; they simply concluded that women with early breast cancer had higher levels than those with advanced disease.

In a study by Ooi et al. (2010), the only randomized controlled trial studying this relationship; it was found that vitamin D deficiency promotes the growth of human breast cancer cells in nude mice.

Ross et al. (2009) investigated the relation between dietary intake of vitamin D and breast cancer risk. Subjects in this study were 2569 women with incident, histologically confirmed breast cancer and 2588 hospital controls. It was concluded that the vitamin D play a significant role in decreasing the risk of breast cancer incidence and progression of breast cancer, and this result is compatible with Abass et al. (2008) that he strongly suggested in increasing vitamin D in daily dietary as protective and anticancer agent.

Another study by Jeong Kim et al. (2011) indicated that vitamin D deficiency correlated with poor outcome for women with breast cancer, this study was conducted in Korea on 310 Korean women treated in Asan Medical Center, to determine the prognostic effects of serum 25-OHD. Expression of estrogen receptor (ER), progesterone receptor (PR), and epidermal growth factor receptor 2 (Her2) were measured using tissue microarrays.

Peppone et al. (2011) conducted a retrospective study on 224 women diagnosed with stage 0-III breast cancer. It was concluded that the deficiency of vitamin D among women with breast cancer affect the prognosis of breast cancer and affect the patients quality of life.

Jacobs et al. (2012) did a prospective cohort study on 3085 as a subject group and 512 as a control group to evaluate the relation between dietary, supplemental, and total vitamin D intake and recurrent breast cancer. Although they stated that Vitamin D intake was not related to breast cancer recurrence overall; they did find a link between vitamin D levels and breast cancer recurrence for premenopausal women.

Yao et al. (2011) also conducted a study on 579 women with incident breast cancer and 574 controls matched on age and time of blood draw enrolled in the Roswell Park Cancer Institute from 2003 to 2008. It was also found that the relationship between vitamin D levels and risk of breast cancer was strongest among premenopausal women.

Anderson et al. (2010) conducted study on breast cancer cases aged 25-74 years old to evaluate the associations and potential interaction between vitamin D and calcium (from food and supplements) and breast cancer risk. An inverse relationship between vitamin D supplementation and breast cancer risk was concluded.

## 3. Conclusion

A comprehensive search of several electronic databases was conducted in PubMed, MEDLINE, CINAHL, In addition, used the web search engine “Google” for abstracts from 1948 to 2012. Key words used included breast cancer, vitamin D, and breast neoplasm. All subheadings were included. To narrow these results, another search was entered with keyword vitamin D. These two searches were then combined and reviewed for appropriateness to the topic, starting with the most recent journal articles and moving backwards chronologically.

The second search was also initiated utilizing the CINAHL data base. Key words included breast cancer and vitamin D. The time frame included 2008 to 2013. Articles were requested from both medical and nursing journals. The third literature search was initiated through the US National Library of Medicine, using Pub Med/ Cochran Collaboration. Key words included vitamin D deficiency related to breast cancer. Appropriate articles were then reviewed, with the latest studies published being chosen. The combined literature search was rewarded with an abundance of articles. To help narrow the findings, articles were excluded according to date, strength of study, and relevance to the research topic.

The rating scale used to rate these studies was the Johns Hopkins Nursing Evidence-Based Practice Model and Guidelines. Several of the individual studies discussed here as it illustrated in Table (2) have been graded as IIIB evidence, and as a whole comprise a level IIIB body of evidence.

**Table 2:** Studies include in the review and their level of evidence

Author(s), year	Design	Sample (N)	Outcomes studied (how measured)	Results	*Quality
Yao et al., 2011	Case-control and case-series study	579 women with primary incident breast cancer 574 controls	Vitamin D levels, in the form of 25OHD, obtained from blood samples via phlebotomy, prior to any treatment	Higher serum levels of 25OHD were associated with reduced risk of breast cancer, with associations strongest for high grade, ER negative or triple negative cancers in premenopausal women.	IIIA
Goodwin et al., 2009	Prospective inception cohort	512 women with early breast cancer, diagnosed 1989-1996	25OHD levels taken from plasma which was collected before initiation of systemic therapy.	Low vitamin D levels had an increased risk of distant recurrence and death.	IIIB
Ooi et al., 2010	RCT	“Subsets” of mice treated concurrently with ostioprotegerin to abrogate bone resorption. One subset (n=7) of mice weaned onto a vitamin D-free diet, which was sustained throughout the study, while the other subset (n=7) were provided with a vitamin D “sufficient“ diet.	Outcomes assessed by repeated radiographic and end-point micro-computed tomography and histologic analysis, as well as the study of breast cancer cell lines, cell proliferation assays, biochemical assays, radiographic quantification of osteolytic lesions, Micro-CT analysis.	“Vitamin D deficiency promotes the growth of human breast cancer cells in the bones of nude mice. These effects are partly mediated through secondary changes in the bone micro environment, along with direct effects of Vitamin D on tumor growth.”	IA
Jacobs et al., 2010	2 study designs were utilized. Prospective cohort study design control study	Prospective cohort study design (n= 3085). Nested case-control study resulted in 512 matched pairs, though some individuals were used as case or control more than once.	Vitamin D intake by a questionnaire outlining their daily eating habits, which included foods consumed, quantity and frequency. Serum vitamin D levels by blood samples were drawn at beginning of study. Questionnaires were also used to collect data on new breast cancer events. These were completed semi-annually.	No relationship between vitamin D levels and breast cancer recurrence was noted in the overall population. However, for pre-menopausal women, a significant inverse relationship between vitamin D levels and recurrence was noted.	IIIB
Anderson et al., 2010	Population based case-control study	4109 cases 4102 controls	Both case and controls completed epidemiological and food frequency questionnaires	No overall association between combined vitamin D and calcium intake and breast cancer risk. However, independent vitamin D supplementation was inversely related to breast cancer risk. Further research needed to investigate high dose supplements of calcium and vitamin D and breast cancer risk.	IIIB
Rossi et al., 2009	Multicenter case–control study	Subjects were 2569 women with incident, histologically confirmed breast cancer and 2588 hospital controls	Cases and controls were interviewed in the hospital by centrally trained interviewers, using food frequency questionnaire (FFQ) was used to assess the patients’ usual diet in the previous 2 years.	Intake of vitamin D >3.57 μg or 143 IU appeared to have a protective effect against breast cancer. The inverse association was consistent across strata of menopausal status.	IIIB
Abbas et al., 2008	Population-based case-control study.	944 Cases 666 controls	Participants complete food frequency questionnaire (FFQ) to assess the association of dietary vitamin D, calcium, and premenopausal breast cancer risk	Strongly suggest a protective effect for post-menopausal breast cancer through a better vitamin D supply as characterized by serum 25(OH)D measurement, with a stronger inverse association in women with low serum 25(OH)D concentrations (<50 nM).	IIIB
Hee Jeong Kim et al. (2010)	Descriptive study	310 Cases	Clinic pathologic data were examined to determine the prognostic effects of serum 25-OHD. Expression of estrogen receptor (ER), progesterone receptor (PR), and epidermal growth factor receptor 2 (Her2) were measured using tissue microarrays.	Vitamin D deficiency may be associated with poor outcomes in patients with luminal-type breast cancer.	IIIB
Peppone et al. (2011)	Descriptive study	224 patients with baseline 25-OH vitamin D values, 126 patients returned in the 8–16 week follow-up window for 25-OH vitamin D reassessment.	Vitamin D deficiency was defined as a 25-OH vitamin D level < 20 ng/ml, insufficiency as 20-31 ng/ml, and sufficiency as ≥32 ng/ml. Bodu miniral density (BMD) was assessed during the period between 3 months before and 6 months following the baseline vitamin D assessment.	Recommended that vitamin D play a significant role in quality of life for women with breast cancer.	IIIB

The studies included in this review utilized various designs, including case-control, case-series, prospective cohort, prospective comparative observational, and randomized controlled trial. Multiple studies used self-report measures to collect data on lifestyle factors such as diet, physical activity, and past medical history. Biophysiological measures were used, including, but not limited to, phlebotomy to measure serum vitamin D levels and analysis of bone structure in mice at a cellular level. Breast cancer status was assessed using a combination of chart reviews, self-reports, and physician reports. With the exception of the study done by Goodwin et al. (2009), methods were clearly stated throughout the studies included here.

It is evident in current science that sufficient levels of vitamin D are beneficial in the fight against breast cancer, but more researches must be done to improve the capability of healthcare providers to prescribe appropriate doses of vitamin D for their patients. The researchers are still unclear on what level of vitamin D is needed to protect against advanced stages of breast cancer.

Adult women, aged 18 and older, should be counseled to take in sufficient amounts of vitamin D each day, with a combination of dietary sources as well as intake from natural sunlight. While the American Dietary Association’s (ADA) guideline promotes food over the use of supplements, dietitians acknowledge that the use of supplements, such as vitamin D, is necessary to fill dietary gaps.

In 1997, the recommended dietary allowance for vitamin D was set at 400 International Units (IUs) for ages 19 to 24; 200 IUs for ages over 24; 400 IUs for ages 50 to 70; and 600 IUs for those over 70 [14]. According to the National Cancer Institute (NCI), a serum level below 15 ng/mL (equivalent to 37.5 nmol/L) is considered inadequate (NCI, 2010). Conclusive research has not been done that allows for a change in these guidelines for current practice.

Educating patients at provider visits with both verbal and written instruction about proper vitamin D supplementation is essential. Before women are started on vitamin D supplementation, an initial blood test to establish a baseline vitamin D level is recommended. Follow-up lab work must continue until the level is within an acceptable range. Continuing education will help to ensure that women understand the importance of maintaining sufficient vitamin D levels.

One of the review limitations is that the studies do not lend itself to level IA evidence. Thus, definitive conclusions cannot yet be made, resulting in a gap in the overall evidence. Current evidence is lacking in dosages of supplementation, and practice lacks a concrete framework for prescribing an appropriate dose of vitamin D based on a patient’s age, risk factors, and other characteristics.

Ooi et al. (2010) recommended that further clinical trials would help to investigate the value of correcting vitamin D deficiency in limiting the progression of breast cancer. More specifically, Yao et al. (2011) called for large prospective studies to be done for temporal causality, while Goodwin et al. (2009) recommended that future randomized trials with adequate power be done that target blood levels of vitamin D.

There is agreement amongst many in the medical community that increased levels of vitamin D are important even as research continues to move in the direction of establishing parameters (Ooi, et al. 2010; Palmieri, et al. 2006). The current environment among both researchers and clinicians is focused on understanding more clearly how vitamin D works metabolically to support and strengthen one’s ability to prevent cancer from occurring or to fight against existing breast cancer. The hope is that with further knowledge of this important process, research might be able to more closely determine the proper levels required to maintain and support a healthy, cancer-free life.
